# The Impact of Perceived Overqualification on Workplace Procrastination: The Role of Public Service Motivation and Perceived Prosocial Impact

**DOI:** 10.3390/bs15050590

**Published:** 2025-04-28

**Authors:** Wenzheng Qiu, Xinyu Dong, Chenhui Liu

**Affiliations:** School of Public Policy and Administration, Xi’an Jiaotong University, Xi’an 710049, China; qiuwenzheng2015@stu.xjtu.edu.cn (W.Q.); liuchenhui@xjtu.edu.cn (C.L.)

**Keywords:** perceived overqualification, workplace procrastination, job boredom, public service motivation, perceived prosocial impact

## Abstract

Workplace procrastination is widespread in the public sector and has severe negative implications. However, research specifically focusing on workplace procrastination among civil servants remains scarce. Drawing on the person–environment (P–E) fit theory and the public service motivation (PSM) fit perspective, this study examines the relationship between perceived overqualification (POQ) and workplace procrastination through job boredom, and further explores how PSM and perceived prosocial impact moderate this relationship. Based on a survey of 363 Chinese civil servants, the findings reveal a positive correlation between POQ and workplace procrastination through job boredom, and this correlation is weaker for civil servants with high PSM than for those with low PSM. A three-way interaction analysis indicates that perceived prosocial impact enhances the ability of PSM to reduce job boredom caused by POQ, which in turn weakens the association between POQ and workplace procrastination. Moreover, when civil servants with high PSM perceive their work as having a high prosocial impact, the association between POQ and workplace procrastination becomes nonsignificant. This study introduces a person–environment interaction perspective for understanding the antecedents of workplace procrastination, underscores the costs of POQ in public organizations, and offers valuable insights for preventing workplace procrastination among civil servants.

## 1. Introduction

Workplace procrastination, defined as the delay of work tasks through engagement in non-work activities without harmful intentions (Metin et al., 2016), is a widespread phenomenon in organizational settings (Edmondson et al., 2024; Hen et al., 2021; Metin et al., 2016). This issue is particularly pronounced in the public sector (Huang et al., 2023), where ambiguous performance evaluation criteria reduce accountability and weaken incentives for timely task completion (X. Liu & Dong, 2012; Mimba et al., 2007). Although often not driven by malicious intent (Metin et al., 2016), workplace procrastination can impair individual well-being (Bilginoğlu & Yalçıntaş, 2020; H. Ma et al., 2021) and organizational efficiency (D’Abate & Eddy, 2007; Haesevoets et al., 2022), with negative and far-reaching societal consequences in the public sector (Sih Darmi & Kusni, 2019). Despite its critical implications, studies specifically addressing civil servants’ workplace procrastination remain scarce. Unlike the private sector, which relies on clear performance metrics to enhance efficiency, the public sector, characterized by high job stability and limited upward mobility (Gao, 2017), faces unique challenges in preventing workplace procrastination. Identifying the antecedents of workplace procrastination in the public sector and exploring targeted prevention strategies are crucial for public organizations.

Previous studies on the antecedents of workplace procrastination have largely centered on individual traits (Huang et al., 2023; Shaw & Choi, 2022) or environmental factors (He et al., 2023; Jian et al., 2024), overlooking the interaction between individuals and their environments. The person–environment (P–E) fit theory emphasizes the importance of compatibility between individuals and their work environment (Kristof-Brown et al., 2005), which is linked to positive outcomes such as higher job performance (Greguras & Diefendorff, 2009; Oh et al., 2014; Uppal, 2021) and enhanced creativity (Choi, 2004). However, when individuals perceive themselves as incompatible with their work environment, such as perceived overqualification (POQ) or underqualification, negative consequences often arise (Luksyte et al., 2011; Sim & Lee, 2018). This study focuses on POQ, a paradoxical situation in which individuals perceive their qualifications as exceeding the demands of their roles (Maynard et al., 2006). Despite the surplus of qualifications, which should ideally enable employees to excel, this surplus often leads to unfavorable effects (S. Liu et al., 2015; Luksyte et al., 2011; B. Ma & Zhang, 2022; McKee-Ryan & Harvey, 2011; Zhao & Ma, 2023). For civil servants, POQ may be particularly pronounced. The public sector’s unique appeal—job stability, social prestige, and opportunities for public service (Ko & Han, 2013)—often attracts potential overqualified candidates, including those with master’s or doctoral degrees (Bao & Zhong, 2024; Shang et al., 2024). Yet, the bureaucratic system, characterized by highly structured roles and standardized tasks (Hansen, 2014; Vigoda-Gadot et al., 2005), limits autonomy and skill utilization, potentially exacerbating POQ. However, studies on the impact of POQ among civil servants remains limited (Bao & Zhong, 2024; Shang et al., 2024), and its relationship with workplace procrastination has yet to be explored.

This study investigates the mediating role of job boredom in the relationship between POQ and workplace procrastination. For employees who feel overqualified, they perceive that their work lacks challenge (Khan et al., 2022), which in turn promotes feelings of boredom (Andel et al., 2022; Bochoridou & Gkorezis, 2024; Sánchez-Cardona et al., 2020). Thus, they seek immediate stimulation, such as cyberloafing or overusing smartphones (Cheng et al., 2020; Peng et al., 2022; Pindek et al., 2018), thereby diverting their attention from ongoing tasks, ultimately resulting in workplace procrastination.

Establishing boundary conditions is essential for mitigating the effects of POQ on workplace procrastination via job boredom. Public service motivation (PSM) is widely regarded as a unique motivational resource in public administration research (Bao & Zhong, 2024; Shim et al., 2017). Individuals with high PSM prioritize societal goals (Perry & Wise, 1990) rather than the desire to utilize their skills, which enables them to better manage the mismatch between their qualifications and job tasks, thereby potentially reducing the impact of POQ on job boredom. Although PSM is often treated as a resource, it also represents a psychological need. The impact of PSM becomes more pronounced when employees with high PSM are able to effectively utilize this motivation in the workplace (Steijn, 2008; Vandenabeele, 2007). In this regard, perceived prosocial impact (PPI)—defined as the extent to which individuals perceive their work as having a positive impact on society (Grant, 2008)—helps meet the needs of individuals with high PSM. Thus, we propose that when employees with high PSM perceive a high level of prosocial impact in their work, the positive relationship between POQ and workplace procrastination mediated by job boredom will be further weakened.

Therefore, this study aims to examine the relationship between POQ and workplace procrastination and investigate the mediating role of job boredom and the moderating roles of PSM and PPI. It makes the following contributions to existing research: First, this study offers a new understanding of the antecedents of workplace procrastination from the person–environment interaction perspective. Second, it highlights the connection between POQ and workplace procrastination, emphasizing that POQ leads to this unintentional but harmful behavior through job boredom. Third, by integrating the literature on POQ and PSM, this research examines the three-way interaction effect of POQ, PSM, and PPI on job boredom and establishes boundary conditions for mitigating workplace procrastination.

## 2. Theory and Hypotheses

### 2.1. The Person–Environment Fit Theory and the Public Service Motivation Fit Perspective

The P–E fit theory depicts “the compatibility between an individual and a work environment that occurs when their characteristics are well matched” (Kristof-Brown et al., 2005, p. 281). When employees perceive a mismatch, they may experience negative work-related outcomes. One common form of misfit is POQ, which occurs when individuals believe their skills, knowledge, and abilities exceed the demands of their job (Erdogan et al., 2011; Maynard et al., 2006). This misfit can leave employees feeling underutilized, which may lead to job boredom and a search for other stimuli, resulting in workplace procrastination.

Individual characteristics play a critical role in shaping psychological responses to P–E misfit (S. Liu et al., 2015; Simon et al., 2019; Wang et al., 2019). For instance, employees with higher justice sensitivity are more likely to experience lower organization-based self-esteem and greater anger toward the employment situation when they perceive themselves as overqualified (S. Liu et al., 2015). PSM is an individual characteristic which is commonly used to explain the attitudes and behaviors of employees in the public sector (Bao & Zhong, 2024; Shim et al., 2017). PSM has been characterized as a sociocentric work orientation (Steijn, 2008), whereas POQ reflects an egocentric orientation. Thus, the adverse impact of POQ may be mitigated when employees have high levels of PSM. 

Scholars of public administration have extended the P–E fit theory to the PSM field, developing the concept of “PSM fit,” which refers to the compatibility between an employee’s PSM and the work environment that occurs when the opportunities in the environment allow the employee’s PSM to be effectively utilized (Steijn, 2008). They argue that only when employees with high PSM can “use” the motivation in the workplace will PSM have a more positive effect (Steijn, 2008; Vandenabeele, 2007). PSM fit has been shown to have significant impacts on employee outcomes in the public sector (Bland et al., 2023; Im et al., 2016; Kroll & Vogel, 2014). PPI—the employees’ perception of the prosocial impact of their work—captures their subjective awareness of how prosocial their work is (Grant, 2008), allowing high-PSM employees to directly see the social value of their work. Previous studies have measured PSM fit by comparing employees’ PSM levels with their evaluation of PPI, achieving a fit when both are high (Steijn, 2008; van Loon et al., 2018). Based on this, we propose that high PSM and high PPI jointly create PSM fit. In this context, employees with high PSM perceive their current work as prosocial, and this prosocial work satisfies their need to serve, allowing them to derive deeper social meaning from their work, thereby further reducing the negative impact of POQ.

Based on the P–E fit theory, this study examines how POQ, a specific form of misfit, impacts workplace procrastination through job boredom. In the context of public administration, it investigates how sociocentric-oriented PSM weakens the effects of egocentric-oriented POQ, and how PSM fit further influences these outcomes.

### 2.2. The Mediating Effect of Job Boredom on the Relationship Between Perceived Overqualification and Workplace Procrastination

Job boredom is described as an uncomfortable condition marked by lack of interest, low arousal, and high dissatisfaction, with a skewed perception of time and difficulties in maintaining attention at work (Harju & Hakanen, 2016; Reijseger et al., 2013; Sánchez-Cardona et al., 2020). It typically arises when employees perceive a mismatch between their needs (e.g., the desire for challenge, skill variety, or meaningful engagement) and the characteristics of their work environment (Gkorezis & Kastritsi, 2017; van Hooff & van Hooft, 2017; van Tilburg & Igou, 2012). Based on the P–E fit theory, while optimal P–E congruence fosters positive organizational outcomes, such as job satisfaction and enhanced performance (Cable & DeRue, 2002), misfit may lead to negative psychological states, particularly when employees experience POQ—the cognitive appraisal that their qualifications exceed job requirements (Erdogan et al., 2011; Maynard et al., 2006).

The psychological impact of POQ is manifested through dual pathways: high-intensity emotional responses (e.g., anger) and low-intensity affective states (e.g., boredom) (Andel et al., 2022; Kim et al., 2021; Peng et al., 2023). For civil servants, specific characteristics such as career reputation and job stability may amplify the importance of low-intensity pathways. These structural factors create a unique environment in which employees tend to recalibrate their expectations when facing discrepancies (Giauque et al., 2012), rather than exhibiting high-intensity negative emotions. This cognitive adjustment process, however, does not address the fundamental psychological need for competence utilization. Employees who are underutilized may experience a lack of stimulation, which can lead to job boredom (Bochoridou & Gkorezis, 2024; Sánchez-Cardona et al., 2020). Thus, we hypothesize the following:

**H1.**  *POQ is positively associated with job boredom.*

Workplace procrastination, defined as the tendency to delay work-related tasks by engaging in activities unrelated to the job (Metin et al., 2016), is a critical challenge to organizational productivity. Job boredom creates a conducive environment for workplace procrastination. When employees feel bored, they instinctively seek alternative stimulation through compensatory activities such as cyberloafing (Pindek et al., 2018) and smartphone overuse (Peng et al., 2022), which help cope with boredom while diverting their attention from work-related tasks (Bench & Lench, 2019). As a result, they postpone their work responsibilities, leading to workplace procrastination.

Previous studies have found that job boredom is a significant predictor of workplace procrastination. For instance, Metin et al. (2016) discovered this association among Dutch white-collar employees. This relationship is further supported by a study conducted among employees in companies across China, highlighting how effective human resource practices can decrease procrastination by addressing boredom (Jian et al., 2024). More recently, Li et al. (2025) found that enhanced human–artificial intelligence interactions reduced boredom, leading to a decrease in procrastination. These studies provide evidence for the link between job boredom and workplace procrastination. Thus, we hypothesize the following:

**H2.**  *Job boredom is positively associated with workplace procrastination.*

Integrating H1 and H2, this study proposes that job boredom mediates the relationship between POQ and workplace procrastination. Specifically, when employees perceive that their qualifications surpass the demands of their job, they develop a negative psychological state—job boredom (Andel et al., 2022; Bochoridou & Gkorezis, 2024; Sánchez-Cardona et al., 2020)—which drives them to seek compensatory stimulation through non-work activities, thereby fostering workplace procrastination (Metin et al., 2016; Peng et al., 2022; Pindek et al., 2018). Therefore, we hypothesize the following:

**H3.**  *Job boredom mediates the positive relationship between POQ and workplace procrastination.*

### 2.3. The Moderating Effect of Public Service Motivation

Individual characteristics play a critical role in shaping psychological responses to P–E misfit (S. Liu et al., 2015; Simon et al., 2019; Wang et al., 2019). For instance, career centrality can intensify the negative effects of POQ (Erdogan et al., 2018), though psychological resilience, on the other hand, can mitigate POQ’s negative consequences (Wang et al., 2019). As an individual characteristic, PSM is a specific form of altruism or prosocial motivation, rooted in values and dispositions shaped by public institutions and their missions (Perry et al., 2010). It is particularly relevant in the context of public sector employees and explains their attitudes and behaviors (Bao & Zhong, 2024; Shim et al., 2017).

PSM, as a sociocentric work orientation, emphasizes the importance of contributing to societal well-being and public service (Steijn, 2008), in contrast to the more egocentric orientation commonly found in POQ. Individuals with high PSM are driven by the desire to make a positive societal impact rather than focusing solely on the personal fulfillment that comes from utilizing their skills. As a result, high-PSM individuals are less likely to experience job boredom due to POQ. Thus, we hypothesize the following:

**H4a.**  *PSM weakens the positive relationship between POQ and job boredom, such that the relationship is weaker when PSM is high rather than low.*

As previously discussed, employees with high PSM are less likely to be affected by the mismatch between their qualifications and job demands, as they are more focused on the broader mission of public service (Perry & Wise, 1990). This sense of purpose may reduce their tendency to experience job boredom, thereby reducing workplace procrastination driven by the need for alternative stimulation. Thus, we hypothesize the following:

**H4b.**  *PSM weakens the positive relationship between POQ and workplace procrastination through job boredom, such that the relationship is weaker when PSM is high rather than low.*

### 2.4. The Moderating Effect of Perceived Prosocial Impact

Building on our previous review of PSM’s moderating role in the POQ–job boredom relationship, we further investigate how PPI interacts with PSM within the PSM fit perspective. Studies on PSM fit suggest that the extent to which employees’ PSM influences their work attitudes and work-related outcomes depends on whether they perceive their work as meaningfully contributing to society (Steijn, 2008; Vandenabeele, 2007). While high-PSM employees are intrinsically motivated to serve the public good, this motivation plays the greatest role when they recognize that their work benefits others (Steijn, 2008). Thus, PPI—employees’ perception of the prosocial impact of their work—acts as a cognitive bridge that enables them to see how their prosocial values are realized in their daily work (Grant, 2008). In this way, high PPI and high PSM together constitute PSM fit, fostering compatibility between employees’ PSM and their work environment. In this state, employees recognize the societal value of their work, which enhances their motivation to work and prevents them from perceiving their work as boring, even when they possess high abilities.

An important consideration is why PPI is particularly relevant for capturing PSM fit in this context. Prior research has identified several factors that contribute to PSM fit, including job characteristics (e.g., high-empowerment practices) and organizational contexts (e.g., high organizational performance, transformational leadership) (Bland et al., 2023; Im et al., 2016; Kroll & Vogel, 2014). While these factors provide conditions for value congruence, they do not directly affect whether high-PSM employees perceive their work as fulfilling their prosocial motivations. Compared to the many objective environmental characteristics that may help employees utilize their PSM, PPI directly captures employees’ subjective awareness that their work benefits others (Grant, 2008), enabling them to see how their efforts contribute to public value.

Although both PSM and PPI are prosocial-oriented constructs, they operate through distinct yet complementary mechanisms. PSM represents a stable dispositional tendency to pursue the public interest (Perry et al., 2010), whereas PPI reflects employees’ perceptions that their work actively contributes to societal well-being (Grant, 2008). We propose that the interaction between PSM and PPI creates a synergistic motivational resource that mitigates the effect of POQ on job boredom. Specifically, when employees with high PSM perceive that their work has a strong prosocial impact, they may reinterpret POQ as an opportunity to apply their skills in ways that benefit society, rather than a trigger for job boredom. Conversely, when either PSM or PPI is low, employees may lack sufficient motivational resources to counteract POQ-induced boredom. For example, low-PSM employees may view high-PPI work as mere obligations rather than job characteristics that meet their needs, while high-PSM employees may struggle to see the societal value of low-PPI work, leading to distress from unfulfilled values. Thus, we hypothesize the following:

**H5a.**  *PSM and PPI jointly moderate the effect of POQ on job boredom, such that when both PSM and PPI are high, the positive relationship between POQ and job boredom is minimized.*

Drawing on the P–E fit theory and the PSM fit perspective, we propose that the interaction between PSM and PPI moderates the relationship between POQ and workplace procrastination through job boredom. As a sociocentric work orientation (Steijn, 2008), PSM mitigates the negative impact of POQ. High PSM shifts employees’ focus on work meaning from personal skill utilization toward seeking social value in their roles (Stritch & Christensen, 2014), while high PPI aligns with the needs of high-PSM employees by reinforcing their perception of work significance (van Loon et al., 2018). When both factors operate at high levels, employees are more likely to establish links between job responsibilities and broader social outcomes, enhancing their perception of work meaningfulness. The PSM fit not only reduces POQ’s impact on job boredom but also diminishes the likelihood of seeking alternative stimuli, thus mitigating workplace procrastination. Therefore, we hypothesize the following:

**H5b.**  *PSM and PPI jointly moderate the indirect effect of POQ on workplace procrastination through job boredom, such that when both PSM and PPI are high, the positive relationship between POQ and workplace procrastination through job boredom is minimized.*

**Figure 1:**
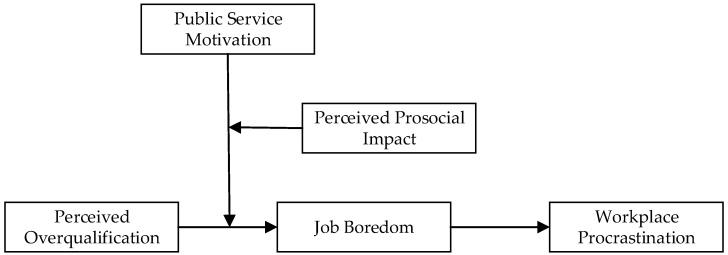
The research model.

The research model is shown in Figure 1.

## 3. Materials and Methods

### 3.1. Sample and Procedure

Data were collected from civil servants in Shaanxi, a western province of China with a mid-level economic development status, recognized as a major hub for education and talent. Civil servants in Shaanxi, like other civil servants across China, are governed by the unified civil service law, which emphasizes their primary duty to serve the public. The questionnaire was distributed with the assistance of the personnel department. Participants were assured that the survey would be used solely for research and that anonymity and confidentiality would be rigorously guaranteed. Written informed consent was obtained from all participants.

To reduce the risk of bias, data collection was conducted in two phases. During the initial phase, participants reported on POQ, PSM, PPI, and job boredom. Two weeks later, the second phase was carried out to gather information on workplace procrastination and demographic characteristics. To ensure that questionnaires completed by the same participant in different phases could be accurately matched, each participant was assigned a unique code in the initial phase, which they were asked to repeat at the top of the second-phase questionnaire.

We distributed 480 questionnaires in each phase and collected 412 and 405 questionnaires in the first and second phases, respectively. By matching codes, 396 paired questionnaires were obtained (pairing response rate = 82.5%). Subsequently, incomplete responses and those containing incorrect answers to validation questions were excluded, leaving a final dataset of 363 valid responses (effective response rate = 75.6%). An initial analysis of the valid responses yielded the following results: male: 192 (52.9%), female: 171 (47.1%); under bachelor’s degree: 10 (2.8%), bachelor’s degree: 247 (68.0%), master’s degree or above: 106 (29.2%). The average age was 32.27 years (*SD* = 7.13).

### 3.2. Measures

The original scales were translated into Chinese using Brislin’s (1970) back-translation procedure. Participants responded on 7-point Likert scales, with options ranging from 1 (strongly disagree) to 7 (strongly agree). The measurements for all the variables are presented in Appendix A, Table A1.

Perceived overqualification. We assessed perceived overqualification using a nine-item scale developed by Maynard et al. (2006), including “I have job skills that are not required for this job.” Cronbach’s α was 0.907.

Public service motivation. The five-item scale developed by Wright et al. (2013) was used to measure civil servants’ PSM, including “Meaningful public service is very important to me.” Cronbach’s α was 0.852.

Perceived prosocial impact. We measured civil servants’ perceived prosocial impact using Grant’s (2008) three-item scale, including “I am very conscious of the positive impact that my work has on others.” Cronbach’s α was 0.840.

Job boredom. We measured job boredom using the four-item scale developed by Pindek et al. (2018), including “I feel bored at my work.” Cronbach’s α was 0.887.

Workplace procrastination. Workplace procrastination was assessed using the six-item scale developed by Kühnel et al. (2016), including “I needlessly delayed finishing jobs, even when they were important.” Cronbach’s α was 0.900.

Control variables. As suggested by prior studies (Cheng et al., 2020; Shang et al., 2024), gender (0 = female, 1 = male), educational level (0 = under bachelor’s degree, 1 = bachelor’s degree, 2 = master’s degree or above), and age (in years) were included.

## 4. Results

### 4.1. Common Method Variance Test and Confirmatory Factor Analysis

We employed two widely used approaches to assess common method variance in the model. First, Harman’s one-factor test showed that five factors were extracted, with the first factor accounting for 26.43% of the variance, which is below the threshold of 40%. Second, a confirmatory factor analysis was performed. As shown in Table 1, the hypothesized five-factor model demonstrated a good fit to the data (χ^2^ [314] = 394.272, χ^2^/*df* = 1.256; RMSEA = 0.027, SRMR = 0.034, CFI = 0.984, TLI = 0.982) and was better than the one-factor model (χ^2^ [324] = 3403.649, χ^2^/df = 10.505, RMSEA = 0.162, SRMR = 0.161, CFI = 0.389, TLI = 0.338) as well as other competitive models. All the above results indicated that common method variance was not a significant concern in this study.

### 4.2. Reliability and Validity

Table 2 presents the results for reliability and validity. All Cronbach’s α and composite reliability values exceed the 0.7 threshold, demonstrating satisfactory reliability, while all average variance extracted (AVE) values surpass the 0.5 threshold, indicating good convergent validity for each construct. In addition, discriminant validity was confirmed as the square root of the AVE for each construct exceeded the correlations with other constructs.

### 4.3. Descriptive Statistics and Correlations

Table 2 presents the means, standard deviations, and correlations of the core variables. The relationships between POQ and job boredom (*r* = 0.356, *p* < 0.001) as well as POQ and workplace procrastination (*r* = 0.234, *p* < 0.001) are both positive and significant. Additionally, there is a positive correlation between job boredom and workplace procrastination (*r* = 0.488, *p* < 0.001).

### 4.4. Hypothesis Testing

Table 3 reports the results of hierarchical regressions on job boredom and workplace procrastination. Hypothesis 1 predicted that POQ has a positive relationship with job boredom. Model 1 included control variables, and Model 2 built upon this by adding POQ. Compared to Model 1, Model 2 showed an increase in R^2^, indicating a stronger explanatory power. The result of Model 2 revealed that POQ was positively related to job boredom (*b* = 0.437, *p* < 0.001), thereby supporting Hypothesis 1.

Hypothesis 2 predicted that job boredom has a positive association with workplace procrastination. The control variables were entered in Model 7, Model 8 built upon this by adding POQ, and Model 9 added job boredom as a predictor of workplace procrastination. R^2^ values increased sequentially across these three models, suggesting that the inclusion of POQ and job boredom improved the explanatory power. In Model 8, POQ was positively related to workplace procrastination (*b* = 0.301, *p* < 0.001). Although the direct effect of POQ on workplace procrastination was no longer significant in Model 9 (*b* = 0.088, *p* = n.s.), job boredom was positively related to workplace procrastination (*b* = 0.487, *p* < 0.001). Therefore, Hypothesis 2 was supported.

Hypothesis 3 predicted that job boredom mediates the relationship between POQ and workplace procrastination. According to Hayes (2009), we performed a bootstrapping analysis with 5000 samples using the PROCESS macro to test the significance of the indirect effect of POQ on workplace procrastination through job boredom. The estimated indirect effect is 0.213, with a 95% confidence interval (CI) of [0.143, 0.289]. Since the 95% CI does not include zero, the indirect effect is considered significant (Hayes, 2009; Preacher & Hayes, 2008). Therefore, Hypothesis 3 was supported.

**Figure 2:**
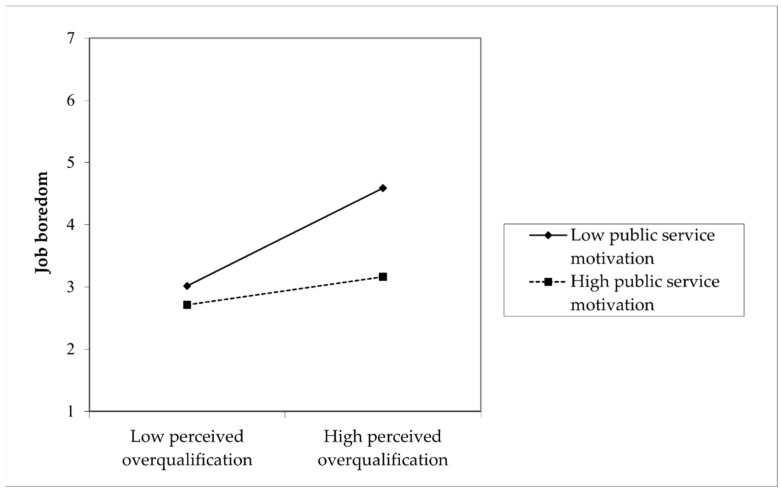
The effect of POQ × PSM on job boredom.

Hypothesis 4a proposed that PSM weakens the effect of POQ on job boredom, such that the effect is weaker when PSM is high rather than low. Model 3 shows that POQ is positively related to job boredom (*b* = 0.445, *p* < 0.001), and the interaction term between POQ and PSM is negatively associated with job boredom (*b* = −0.220, *p* < 0.001). As shown in Figure 2, simple slope analyses confirmed that the effect of POQ on job boredom was stronger when the level of PSM was low (*b* = 0.692, *p* < 0.001) than when it was high (*b* = 0.198, *p* < 0.05). Thus, Hypothesis 4a was supported.

To further examine whether PSM moderated the indirect effect of POQ on workplace procrastination through job boredom, we conducted a bootstrapping analysis with 5000 resamples using the PROCESS macro, in line with the recommendations of Hayes (2009) and Preacher and Hayes (2008). Consistent with prior studies (Chen et al., 2025; Zhou et al., 2024), we assessed the moderated indirect effects when PSM was at one standard deviation (SD) above and below the mean. As shown in Table 4, the effect was stronger when PSM was lower (Effect = 0.337, 95% CI = [0.243, 0.441]) than when it was higher (Effect = 0.096, 95% CI = [0.020, 0.180]). Additionally, there was a significant difference between these effects (diff = −0.241, 95% CI = [−0.362, −0.132]). The results showed that PSM weakened the positive relationship between POQ and workplace procrastination through job boredom. Thus, Hypothesis 4b was supported.

Hypothesis 5a predicted a three-way interaction of POQ, PSM, and PPI on job boredom. To test this hypothesis, we sequentially added variables starting with Model 1, which included control variables. The main predictors (POQ, PSM, and PPI) were then added to Model 4, followed by three two-way interactions in Model 5, and finally, a three-way interaction in Model 6. Table 3 reports the results. The increase in R^2^ suggests that the model becomes better at explaining job boredom as more predictors and interaction terms are added. As shown in Model 6, POQ was positively related to job boredom (*b* = 0.475, *p* < 0.001), and the three-way interaction of POQ, PSM, and PPI was negatively associated with job boredom (*b* = −0.127, *p* < 0.01).

**Figure 3:**
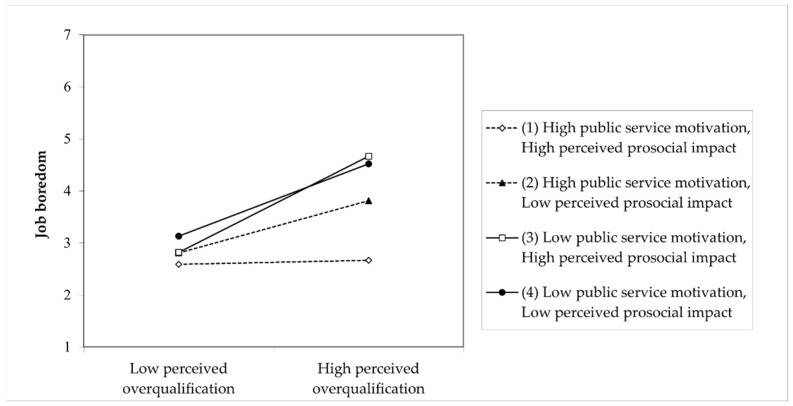
The effect of POQ × PSM × PPI on job boredom.

To understand the nature of the three-way interaction, we plotted the relationship between POQ and job boredom, as recommended by Aiken and West (1991) and Dawson and Richter (2006) (see Figure 3). When PSM and PPI were both high, the effect of POQ on job boredom was the weakest. Following Dawson and Richter (2006), simple slope analyses were performed to examine the significant three-way interaction effect. The slopes of high PSM–low PPI (*b* = 0.441, *p* < 0.001), low PSM–high PPI (*b* = 0.812, *p* < 0.001), and low PSM–low PPI (*b* = 0.610, *p* < 0.001) are significant and positive. Only for the high PSM–high PPI group is the slope not significant (*b* = 0.036, *p* = 0.731).

Following Dawson and Richter (2006), we conducted a slope difference test for the three-way interaction effect. As shown in Table 5, the slope for the high PSM–high PPI group was significantly different from that of the high–low (*t* = −2.575, *p* < 0.05), low–high (*t* = −4.827, *p* < 0.001) and low–low (*t* = −3.855, *p* < 0.001) groups; the slope for the high PSM–low PPI group was different from that for the low PSM–high PPI group (*t* = −2.191, *p* < 0.05). Thus, the slope difference results, combined with the significant three-way interaction coefficient shown in Model 6 and the results of simple slope analyses, supported Hypothesis 5a.

To further examine whether PSM and PPI jointly moderated the indirect effect of POQ on workplace procrastination through job boredom, we employed a bootstrapping analysis. This method is preferred due to its superior statistical power and ability to estimate confidence intervals for mediation paths at varying levels of moderators (Edwards & Lambert, 2007). It has been used in previous studies to investigate the indirect effects of three-way interactions (Feng et al., 2024; Quade et al., 2019). Following Hayes’ (2009) recommendations, we conducted 5000 bootstrap resamples using the PROCESS macro to ensure the stability of parameter estimates. Consistent with the operationalization used by Quade et al. (2019), we assessed the significance of the conditional process by examining whether the 95% CI for the four combinations of PSM and PPI—both above the mean by one SD, both below the mean by one SD, PSM above the mean by one SD with PPI below the mean by one SD, and PSM below the mean by one SD with PPI above the mean by one SD—included zero. An interval that does not contain zero indicates statistical significance for that specific condition (Hayes, 2009; Preacher & Hayes, 2008). As shown in Table 6, the effect of POQ on workplace procrastination through job boredom was positive and significant for the high PSM–low PPI (Effect = 0.215, 95% CI = [0.098, 0.345]), low PSM–high PPI (Effect = 0.396, 95% CI = [0.277, 0.534]), and low PSM–low PPI groups (Effect = 0.297, 95% CI = [0.184, 0.413]), while the effect was the weakest and not significant when both PSM and PPI were high (Effect = 0.017, 95% CI = [−0.069, 0.106]). Thus, Hypothesis 5b was supported.

## 5. Discussion

Based on the P–E fit theory and the PSM fit perspective, this study examines the relationship between POQ and workplace procrastination through job boredom among civil servants, focusing on the moderating roles of PSM and PPI.

First, job boredom mediates the relationship between POQ and workplace procrastination. Civil servants who perceive themselves to be overqualified may adjust their expectations due to the potential benefits of a public-sector career (Giauque et al., 2012), such as social prestige, job stability, and potential service opportunities (Ko & Han, 2013), rather than experiencing high-intensity emotions. However, this cognitive adjustment process does not fully address the underlying need for skill utilization and meaningful work, leading to a state of understimulation, which results in boredom. This emotional state prompts employees to seek stimulation, such as cyberloafing or overusing smartphones (Peng et al., 2022; Pindek et al., 2018). Their attention is diverted from work-related tasks, resulting in workplace procrastination.

Second, PSM weakens the positive relationship between POQ and job boredom, as well as the relationship between POQ and workplace procrastination through job boredom. Individuals with high PSM are often motivated by values such as altruism, compassion, and civic duty (Perry, 1996), which help reduce egocentric concerns. However, the effect of POQ on workplace procrastination is weakened but remains significant when PSM is high, suggesting that while PSM weakens the impact of POQ on job boredom, it does not fully eliminate this effect.

Third, when both PSM and PPI are high, POQ no longer has a significant effect on job boredom, nor an indirect effect on procrastination through job boredom. PPI plays a crucial role in facilitating PSM fit by providing employees with clear evidence that their work positively impacts others. Unlike objective organizational factors (e.g., leadership style or workplace culture), PPI directly influences employees’ perception that their efforts contribute to society—making it a powerful factor in reinforcing PSM’s positive effects. When both PSM and PPI are high, employees gain a strong sense of fulfillment, which helps them overcome job boredom triggered by feeling overqualified. In addition to the strong evidence confirming that the effect of POQ on job boredom is minimized when both PSM and PPI are high, this study also found that POQ’s impact on job boredom is weaker in the high PSM–low PPI group compared to the low PSM–high PPI group. This suggests that low-PSM employees may view high-PPI tasks as mere obligations rather than as job characteristics that meet their needs, indicating that for individuals with low PSM, engaging in prosocial service is less effective in combating job boredom. These findings highlight that recruiting individuals with high PSM or enhancing civil servants’ PSM, as well as fulfilling their need to contribute to society, are crucial for minimizing the harmful consequences of POQ.

### 5.1. Theoretical Contributions

This study has important theoretical contributions. First, this study provides a new perspective for explaining the far-reaching phenomenon of workplace procrastination. Previous studies on workplace procrastination have considered environmental characteristics (e.g., humble leadership, developmental human resource practice) (He et al., 2023; Jian et al., 2024) or individual traits (e.g., personality) (Huang et al., 2023; Shaw & Choi, 2022) as separate antecedents. This study, based on the P–E fit theory and the PSM fit perspective, examines how individuals’ perception that their abilities exceed job demands affects workplace procrastination, and further explores how this relationship changes when there is a match in PSM among civil servants.

Second, while POQ has received growing attention in public management studies (Bao & Zhong, 2024; Shang et al., 2024), this study reveals its impact on a prevalent yet underexplored outcome in the public sector—workplace procrastination. When civil servants encounter a work environment that they perceive as wasting their talents, they may respond by lowering their expectations of the job or the work environment (Giauque et al., 2012). Although this may not result in intense counterproductive behaviors, our study suggests that this perception of overqualification leads to workplace procrastination through job boredom. Our findings thus offer a new perspective on the organizational costs of POQ in public administration, shifting the focus from overt counterproductive behaviors to more subtle declines in productivity such as workplace procrastination.

Third, this study reveals the moderating roles of PSM and PPI in the relationship between POQ and job boredom. Although PSM can reduce the positive relationship between POQ and job boredom, it cannot eliminate the effect. Building on the previous study that found that PSM can mitigate the detrimental effects of POQ among public sector employees (Bao & Zhong, 2024), our study adopts the PSM fit perspective and treats PSM as a psychological need, suggesting that when individuals with high PSM perceive their motivation as being effectively utilized, the adverse effects of POQ can be minimized or even eliminated. This study challenges the assumption that PSM alone can give meaning to daily work (Bao & Zhong, 2024). Instead, we propose that PSM is a prosocial motivation that requires a supportive environment for fulfillment. It is the combination of high PSM and high PPI that shapes the perception of meaningful work, thereby eliminating the impact of POQ on job boredom.

### 5.2. Practical Implications

Workplace procrastination among civil servants not only impacts individual well-being and organizational efficiency but also has broader negative societal consequences. Drawing on the P–E fit theory and the PSM fit perspective, this study provides practical insights for addressing workplace procrastination in the public sector.

First, public organizations should emphasize the importance of person–job fit. One effective strategy would be for them to collaborate with universities, providing students with internship opportunities within the public sector. These internships would allow students to gain firsthand experience of the realities of civil service work, helping them develop a clear understanding of public-sector roles. This would prevent the mismatch between personal expectations and actual job requirements that often leads to boredom and procrastination after blindly applying for positions. Moreover, public organizations should provide as much detailed information about the position as possible in their recruitment announcements, thereby giving candidates a valuable reference for their application. Additionally, public managers should regularly assess employees’ performance and capabilities, making dynamic adjustments to specific job tasks to ensure a better match between employees’ qualifications and the demands of their roles.

Second, given that job boredom is a key mechanism linking POQ to workplace procrastination, public organizations should also focus on job design to address this issue. Reducing unnecessary rules and procedures, and developing programs to automate repetitive tasks will free up more time for civil servants to engage in meaningful work and focus on the more creative and impactful aspects of their roles. Moreover, offering opportunities for job rotation would help prevent boredom by ensuring employees do not remain in the same routine tasks for extended periods.

Third, public organizations should recognize the importance of PSM in shaping civil servants’ attitudes and behaviors. They should incorporate objective testing tools and subjective interviews that assess candidates’ PSM as a key component of the recruitment results, aiming to select individuals who are eager to serve in the public sector. In addition, organizations should cultivate service-oriented values by publicly recognizing and rewarding individuals who excel in service, and inviting accomplished public service practitioners to share their experiences.

Finally, public organizations should provide high-PSM employees with more opportunities for public service to utilize their talents, thereby enabling them to experience the societal value of their work, which boosts their motivation to engage in their roles. Public managers should streamline workflows and use new technologies to assist in handling repetitive tasks, thus giving employees time to provide public service and contribute ideas. Capable civil servants often identify various issues in their daily work, yet some of these may be temporarily unsolvable due to institutional constraints or practical limitations. Such situations will reduce their enthusiasm. Therefore, it is essential to establish an internal proposal platform for civil servants, encouraging them to propose solutions for challenges in public service. Proposals deemed valuable after evaluation by experts and leaders should receive financial support or be further developed through cross-departmental collaborations organized by senior leadership. This approach not only allows civil servants with high POQ to utilize their qualifications but also enables high-PSM employees to witness the societal impact of their work, reducing job boredom and workplace procrastination and enhancing overall organizational efficiency.

### 5.3. Limitations and Future Directions

First, while this study reveals a positive correlation between POQ and workplace procrastination through job boredom by drawing on the P–E fit theory, the other form of misfit—perceived underqualification (PUQ) (Sim & Lee, 2018)—has not been examined in relation to workplace procrastination. For employees who perceive themselves as underqualified, they may experience a heightened fear of failure, which has been shown to be related to procrastination (Danne et al., 2024). This mechanism is distinct from POQ’s boredom-driven procrastination mechanism. Additionally, the role of PSM may differ between employees with high POQ and those with high PUQ. While our findings show that PSM mitigates POQ’s detrimental effects by helping employees focus on meaningful public goals, it might intensify pressure for employees with high PUQ. High-PSM employees facing skill gaps could experience greater anxiety about meeting public expectations, which may lead to workplace procrastination caused by PUQ. Future studies should compare how POQ and PUQ trigger procrastination and examine the role of PSM in this process.

Second, this study selected employees’ perceptions of prosocial impact as the factor that matches the needs of high-PSM employees, given that this type of influence is the most direct. Nevertheless, to better guide future practices towards meeting the needs of high-PSM employees, it would be beneficial to explore more specific forms of support, such as providing opportunities for employees to interact with the public or granting them greater job autonomy.

Third, although this study collected data in two stages to reduce concerns about common method bias, it is essentially a cross-sectional study. Future research could adopt a longitudinal design to strengthen causal inferences.

Fourth, research on POQ among civil servants has received less attention compared to the extensive research on its effects on private sector employees. Future studies could explore the differences in the causes and outcomes of POQ between civil servants and private sector employees.

## 6. Conclusions

Workplace procrastination is a prevalent issue in the public sector, often carrying severe negative implications. However, research on the antecedents of workplace procrastination among civil servants remains limited. Drawing on the P–E fit theory and the PSM fit perspective, this study explored the relationship between POQ and workplace procrastination, taking the mediating role of job boredom and the moderating roles of PSM and PPI into consideration.

The findings reveal that job boredom mediates the positive association between POQ and workplace procrastination, and this relationship is weaker for civil servants with higher PSM compared to those with lower PSM. However, high PSM alone cannot eliminate job boredom caused by POQ. A three-way interaction analysis indicates that PPI enhances the ability of PSM to reduce job boredom caused by POQ, and to weaken the indirect effect of POQ on workplace procrastination. Moreover, when civil servants with high PSM perceive their work as having a high prosocial impact, the association between POQ and workplace procrastination becomes nonsignificant.

This study introduces person–environment interaction as a new perspective for exploring the antecedents of workplace procrastination. Unlike most previous studies that focus on the high-intensity emotions and counterproductive behaviors generated by employees’ POQ in the private sector, this study highlights a more subtle cost of POQ in the public sector—namely, increased workplace procrastination. Furthermore, this study investigates the boundary conditions that mitigate the impact of POQ on workplace procrastination, providing valuable insights for preventing workplace procrastination in the public sector.

## Figures and Tables

**Table 1:** Results of confirmation factor analysis.

Models	χ^2^	*df*	χ^2^/*df*	RMSEA	SRMR	CFI	TLI
Five-factor model (POQ, PSM, PPI, JB, WP)	394.272	314	1.256	0.027	0.034	0.984	0.982
Four-factor model (POQ, PSM + PPI, JB, WP)	853.250	318	2.683	0.068	0.068	0.894	0.883
Four-factor model (POQ, PSM, PPI, JB + WP)	996.176	318	3.133	0.077	0.076	0.865	0.852
Three-factor model (POQ, PSM + PPI, JB + WP)	1451.671	321	4.522	0.099	0.096	0.776	0.755
Two-factor model (POQ + PSM + PPI, JB + WP)	2183.813	323	6.761	0.126	0.135	0.631	0.599
One-factor model (POQ + PSM + PPI + JB + WP)	3403.649	324	10.505	0.162	0.161	0.389	0.338

Note: N = 363. “+” represents the combination of factors. POQ = perceived overqualification; PSM = public service motivation; PPI = perceived prosocial impact; JB = job boredom; WP = workplace procrastination.

**Table 2:** Reliability, validity, descriptive statistics, and correlations.

Variables	Mean	SD	α	CR	AVE	1	2	3	4	5
1. Perceived overqualification	4.314	1.137	0.907	0.909	0.525	0.725				
2. Public service motivation	5.177	1.126	0.852	0.856	0.544	−0.031	0.737			
3. Perceived prosocial impact	5.518	1.061	0.840	0.842	0.642	0.092	0.069	0.801		
4. Job boredom	3.376	1.426	0.887	0.889	0.666	0.356 ***	−0.324 ***	−0.127 *	0.816	
5. Workplace procrastination	3.715	1.529	0.900	0.903	0.610	0.234 ***	−0.114 *	−0.102	0.488 ***	0.781

Note: N = 363. CR = composite reliability; AVE = average variance extracted. The square roots of AVE values are presented on the diagonal, and Pearson correlation coefficients are presented in the lower triangle. * *p* < 0.05, *** *p* < 0.001.

**Table 3:** Results of hierarchical regressions.

Variables	Job Boredom						Workplace Procrastination		
	Model 1	Model 2	Model 3	Model 4	Model 5	Model 6	Model 7	Model 8	Model 9
Intercept	2.727 ***	2.960 ***	3.091 ***	3.026 ***	3.155 ***	3.110 ***	2.833 ***	2.993 ***	1.551 ***
Control									
Gender	−0.238	−0.205	−0.077	−0.062	−0.027	0.001	−0.234	−0.212	−0.112
Age	0.015	0.008	−0.001	0.002	−0.001	−0.002	0.020	0.016	0.012
Education	0.240	0.200	0.283 *	0.243	0.223	0.260 *	0.283	0.255	0.158
Main Predictors									
POQ		0.437 ***	0.445 ***	0.444 ***	0.457 ***	0.475 ***		0.301 ***	0.088
PSM			−0.383 ***	−0.389 ***	−0.370 ***	−0.362 ***			
PPI				−0.176 **	−0.188 **	−0.180 **			
Two-way Interactions									
POQ ×PSM			−0.220 ***		−0.210 ***	−0.210 ***			
POQ × PPI					−0.049	−0.048			
PSM × PPI					−0.136 *	−0.125 *			
Three-way Interaction									
POQ × PSM × PPI						−0.127 **			
Mediator									
Job boredom									0.487 ***
R^2^	0.017	0.137	0.271	0.251	0.303	0.318	0.020	0.070	0.248
ΔR^2^	—	0.120 ***	0.134 ***	0.234 ***	0.052 ***	0.014 **	—	0.050 ***	0.178 ***
		(2 vs. 1)	(3 vs. 2)	(4 vs. 1)	(5 vs. 4)	(6 vs. 5)		(8 vs. 7)	(9 vs. 8)

Note: N = 363. Coefficients are unstandardized. * *p* < 0.05, ** *p* < 0.01, *** *p* < 0.001.

**Table 4:** Moderated indirect effects of PSM at different levels.

Conditional Indirect Relationships	Perceived Overqualification → Job Boredom → Workplace Procrastination		
	Effect	BootSE	Bootstrap 95% CI
High public service motivation (+1 SD)	0.096	0.040	[0.020, 0.180]
Low public service motivation (−1 SD)	0.337	0.050	[0.243, 0.441]
Difference	−0.241	0.058	[−0.362, −0.132]

Note: N = 363. Number of bootstrap samples = 5000. BootSE = bootstrap standard error. CI = confidence interval. SD = standard deviation.

**Table 5:** Results of slope difference test for the three-way interaction.

Slope Difference		Job Boredom	
		*t*	*p*
High PSM (+1 SD), high PPI (+1 SD)	High PSM (+1 SD), low PPI (−1 SD)	−2.575	0.010
	Low PSM (−1 SD), high PPI (+1 SD)	−4.827	0.000
	Low PSM (−1 SD), low PPI (−1 SD)	−3.855	0.000
High PSM (+1 SD), low PPI (−1 SD)	Low PSM (−1 SD), high PPI (+1 SD)	−2.191	0.029
	Low PSM (−1 SD), low PPI (−1 SD)	−1.048	0.295
Low PSM (−1 SD), high PPI (+1 SD)	Low PSM (−1 SD), low PPI (−1 SD)	1.274	0.203

Note: N = 363.

**Table 6:** Moderated indirect effects of PSM and PPI at different levels.

Conditional Indirect Relationships	Perceived Overqualification → Job Boredom → Workplace Procrastination		
	Effect	BootSE	Bootstrap 95% CI
High PSM (+1 SD), high PPI (+1 SD)	0.017	0.045	[−0.069, 0.106]
High PSM (+1 SD), low PPI (−1 SD)	0.215	0.063	[0.098, 0.345]
Low PSM (−1 SD), high PPI (+1 SD)	0.396	0.065	[0.277, 0.534]
Low PSM (−1 SD), low PPI (−1 SD)	0.297	0.058	[0.184, 0.413]

Note: N = 363. Number of bootstrap samples = 5000.

## Data Availability

The data supporting this study can be obtained from the corresponding author upon reasonable request.

## Abbreviations

The following abbreviations are used in this manuscript:

P–E fit	Person–environment fit
PSM	Public service motivation
POQ	Perceived overqualification
PPI	Perceived prosocial impact
JB	Job boredom
WP	Workplace procrastination
RMSEA	Root-mean-square error of approximation
SRMR	Standardized root-mean-square residual
CFI	Comparative fit index
TLI	Tucker–Lewis index
CR	Composite reliability
AVE	Average variance extracted
CI	Confidence interval
SD	Standard deviation

## Appendix A

**Table A1:** Measurement items.

Scales	Items
Perceived Overqualification	1. My job requires less education than I have.
	2. The work experience that I have is not necessary to be successful on this job.
	3. I have job skills that are not required for this job.
	4. Someone with less education than myself could perform well on my job.
	5. My previous training is not being fully utilized on this job.
	6. I have a lot of knowledge that I do not need in order to do my job.
	7. My education level is above the education level required by my job.
	8. Someone with less work experience than myself could do my job just as well.
	9. I have more abilities than I need in order to do my job.
Public Service Motivation	1. Meaningful public service is very important to me.
	2. I am often reminded by daily events about how dependent we are on one another.
	3. Making a difference in society means more to me than personal achievements.
	4. I am prepared to make enormous sacrifices for the good of society.
	5. I am not afraid to go to bat for the rights of others even if it means I will be ridiculed.
Perceive Prosocial Impact	1. I am very conscious of the positive impact that my work has on others.
	2. I am very aware of the ways in which my work is benefiting others.
	3. I feel that I can have a positive impact on others through my work.
Job Boredom	1. At work, time goes by very slowly.
	2. I feel bored at my job.
	3. There are long periods of boredom on the job.
	4. At work, I spend my time aimlessly.
Workplace Procrastination	1. I needlessly delayed finishing jobs, even when they were important.
	2. I delayed making tough decisions.
	3. I was an incurable time waster.
	4. I was a time waster but I couldn’t seem to do anything about it.
	5. I promised myself I’ll do something and then dragged my feet.
	6. I got stuck in neutral even though I knew how important it was to get started.
